# Development of Unconventional T Cells Controlled by MicroRNA

**DOI:** 10.3389/fimmu.2019.02520

**Published:** 2019-10-23

**Authors:** Samantha J. Winter, Andreas Krueger

**Affiliations:** Institute for Molecular Medicine, Goethe-University Frankfurt, Frankfurt am Main, Germany

**Keywords:** thymus, T cell, MAIT cell, NKT cell, Treg cell, γδT cell, microRNA, miR-181

## Abstract

Post-transcriptional gene regulation through microRNA (miRNA) has emerged as a major control mechanism of multiple biological processes, including development and function of T cells. T cells are vital components of the immune system, with conventional T cells playing a central role in adaptive immunity and unconventional T cells having additional functions reminiscent of both innate and adaptive immunity, such as involvement in stress responses and tissue homeostasis. Unconventional T cells encompass cells expressing semi-invariant T cell receptors (TCRs), such as invariant Natural Killer T (iNKT) and Mucosal-Associated Invariant T (MAIT) cells. Additionally, some T cells with diverse TCR repertoires, including γδT cells, intraepithelial lymphocytes (IEL) and regulatory T (Treg) cells, share some functional and/or developmental features with their semi-invariant unconventional counterparts. Unconventional T cells are particularly sensitive to disruption of miRNA function, both globally and on the individual miRNA level. Here, we review the role of miRNA in the development and function of unconventional T cells from an iNKT-centric point of view. The function of single miRNAs can provide important insights into shared and individual pathways for the formation of different unconventional T cell subsets.

## Unconventional T Cells

T cells make up a central part of the adaptive immune system. Certain T cell populations, frequently referred to as unconventional T cells, share functional profiles of both innate and adaptive immunity. These innate-like T cells have the capacity to rapidly respond to non-cognate stimulation by releasing large amounts of cytokines on top of their characteristic T-cell receptor-mediated functions. In addition to direct anti-pathogenic actions, innate-like cells have also been implicated in stress responses and tissue homeostasis. Invariant Natural Killer T cells (iNKTs), certain γδT cell populations, Mucosal-Associated Invariant T (MAIT) cells and Intraepithelial Lymphocytes (IELs) constitute key variants of unconventional T cells (1–4). Thymus-derived regulatory T (tTreg) cells share at least some developmental features with unconventional innate-like T cells and are therefore discussed here as well (5, 6).

Broadly, unconventional T cells are generated through two major developmental pathways. The vast majority of γδT cells and a smaller percentage of IELs and iNKT cells are derived from CD4^−^CD8^−^ co-receptor double negative (DN) thymocytes (7, 8). In contrast, most IELs, iNKT cells, MAIT cells, and tTreg cells are formed after passing through the more mature CD4^+^CD8^+^ double-positive (DP) thymocyte stage in a process termed agonist selection (7, 9). In this review, we focus on the latter subset of cells.

iNKT cells are characterized by their semi-invariant T cell receptor (TCR), which is comprised of an invariant Vα14Jα18 TCRα chain in mouse (Vα24Jα18 in human) and TCRβ chains with a bias for Vβ8, Vβ7, and Vβ2 (10, 11). These TCRs recognize glycolipid antigen in the context of the non-classical MHC molecule CD1d (12). In the thymus, iNKT-cell development can be divided into four consecutive stages (S0–3) based on differential expression of CD24, CD44, and NK1.1 (13, 14). Of note, S2 cells (CD44^+^NK1.1^−^) have the capacity to leave the thymus and enter peripheral tissues, whilst S3 cells (NK1.1^+^) are mostly thymus-resident (15). Functionally, iNKT cells can be subdivided into NKT1, NKT2, and NKT17 subsets based on prototypical cytokine and transcription factor signatures (IFNγ and T-bet for NKT1, IL-4 and IL-13 for NKT2 and IL-17 and RORγt for NKT17) (16).

In mice, MAIT cells are considerably less abundant than iNKT cells (with the opposite being the case in humans). The semi-invariant TCR of MAIT cells consists of a Vα19Jα33 TCRα chain in mice (Vα7.2Jα33 in humans) and a biased TCRβ chain usage (17, 18). MAIT cell TCRs recognize vitamin B derivatives in the context of the MHC-class I related molecule 1 (MR1) (19). Similar to iNKT cells, intrathymic development of MAIT cells can be divided into intermediates based on expression of the surface markers CD24 and CD44 (20). There is evidence that stage 2 (S2) MAIT cells exit the thymus, whereas S3 cells represent recirculating or resident cells. Further similarities to iNKT cells exist, with the description of MAIT1 and MAIT17 cells, which preferentially reside within defined tissues and possess more similar transcriptome profiles to NKT1 and NKT17 cells, respectively, than they do to MAIT cells of different tissues (21). Whereas, ratios of MAIT17 and MAIT1 cells are skewed in favor of MAIT17 cells in virtually every organ, the opposite is the case for NKT17 and NKT1 cells. However, both MAIT17 and NKT17 cells are preferentially enriched in lung, skin, and the colonic lamina propria. Notably, no MAIT2 cells have been described, although production of IL-13 has been demonstrated in long-term stimulated human MAIT cells (22). Development of all iNKT and MAIT cell lineages depends on expression of the transcription factor PLZF encoded by *Zbtb16* (20, 23, 24). Both iNKT cells and MAIT cells are selected on DP thymocytes rather than epithelial cells. These cells also provide essential signals to iNKT cells through homotypic SLAM family receptor interactions, albeit it remains to be established whether MAIT cells are equally dependent on such signals (25, 26). Recently, a minor subset of MAIT cells has been described that is selected on thymic epithelial cells rather than thymocytes, retains a naive phenotype and is independent of SLAM signaling (27). Despite it being well-established that iNKT cells are derived from cells that have received strong TCR signals, requirement of such signals remains a major open question in MAIT-cell development. Lack of efficient MAIT-cell development in germ-free mice has suggested that MAIT cells are selected on foreign antigen (20, 28). In fact, it has been demonstrated that DP thymocytes are capable of rapidly presenting exogenously administered antigen, resulting in recovery of MAIT cells (28).

IELs constitute one of the most numerous lymphocyte populations in the body. They support intestinal tissue homeostasis and the integrity of the epithelial barrier between gut lumen and the body. IELs comprise conventional αβT cells, γδT cells as well as unconventional αβT cells (29). The latter are characterized by lack of the co-receptors CD4 and CD8αβ and expression of the CD8αα homodimer. CD8αα IELs rely on strong TCR signals during development and are partially autoreactive (30, 31). Recently, two distinct IEL precursor populations have been identified (31). However, their interrelatedness and developmental fate remain elusive.

tTreg cells have a diverse TCR repertoire, which partially displays characteristics of autoreactivity (32). Consistently, intrathymic development of most tTreg cells requires strong signals through their TCRs (5). As a consequence, additional signals via CD28 and IL-2 are required to prevent emerging tTreg cells from clonal deletion. Development of tTreg cells can be characterized by successive expression of the signature transcription factor Foxp3 and the IL-2 receptor alpha chain, CD25. Some tTreg cells emerge from precursors expressing first Foxp3 and then CD25 and some tTreg cells are generated through a CD25^+^Foxp3^−^ intermediate (33–36). Recently, it has been suggested that tTreg cells have distinct functions depending on their developmental origin (37).

Although basic principles of T-cell development are shared between mice and humans, there are some fundamental differences in regards to unconventional T cells. For instance, the αβ vs. γδ lineage decision in humans has distinct requirements for NOTCH signals when compared to the murine system (38, 39). Moreover, it has recently been suggested that the timing of agonist selection in humans might differ from that in mice (40). Another notable difference between mice and humans is the relative abundance of iNKT and MAIT cells. In mice iNKT cells are the predominant population, whereas in humans MAIT cells are much more abundant. The underlying reasons for this difference remain unknown. In this review, we focus on the murine system because genetic models have so far played a substantial role in uncovering cell-type specific microRNA (miRNA) function. Individual miRNAs discussed here are conserved between the two species. However, miRNA binding sites might show a lower degree of conservation. Therefore, it might be interesting to explore whether species-specific differences of miRNA-mediated gene regulation contribute to differences between human and murine unconventional T cells.

Despite some significant differences, it is evident that unconventional T cells share multiple developmental principles, such as the requirement for strong TCR signals and a second signal conferring protection from cell death (such as CD28 and IL-2 for Treg cells and SLAM family receptor signaling for iNKT cells). Of note, both commonalities remain hypothetical in the case of MAIT cells (41). Here, we review the role of miRNAs in development of unconventional T cells. Emerging roles for miRNAs may highlight further common features and differences between different types of unconventional T cells.

## Role of miRNAs in the Development of Unconventional T Cells

miRNAs are a form of small non-coding RNA, which principally act as post-transcriptional regulators of gene expression. Upon maturation from precursors, individual miRNAs are incorporated into RNA-induced silencing complexes (RISC), binding to and destabilizing mRNA transcripts, blocking translation and promoting mRNA decay (42). The ability to simultaneously modulate hundreds of mRNA transcripts at a relatively low metabolic cost to a cell is a major advantage of miRNA-mediated gene regulation (43). Conditional deletion of critical components of the miRNA processing machinery, such as Drosha, Dgcr8, and Dicer, which results in loss of the vast majority of miRNAs, has revealed a particular sensitivity in the development of unconventional αβT cells to general miRNA deficiency. In contrast, intrathymic γδT-cell numbers remained comparatively unaffected by loss of all miRNAs, thus ultimately resulting in elevated overall frequencies due to preferential loss of αβ lineage thymocytes (44).

**Figure 1:**
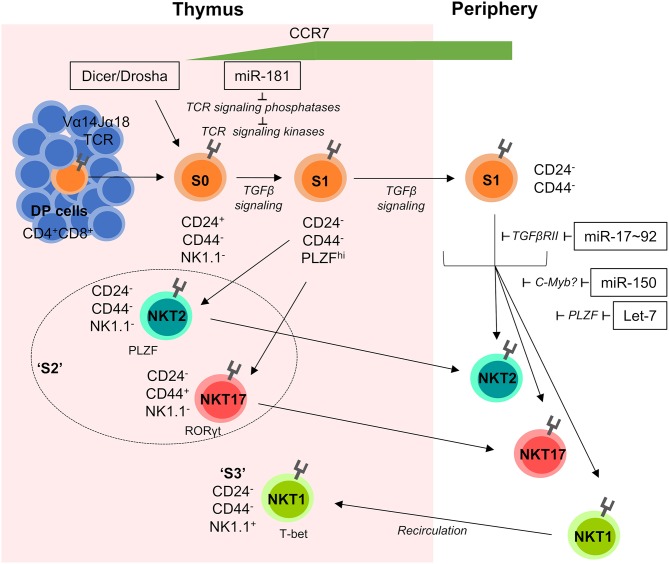
miRNA-mediated control of iNKT-cell development. iNKT cells are selected on CD1d-expressing DP cells in the thymus. Subsequently they progress through distinct developmental stages S0–S3 characterized by their dynamic expression of CD24, CD44, NK1.1, and the transcription factors PLZF, RORγt, and T-bet. Early stage iNKT cells are sensitive to the loss of total miRNAs in Dicer and/or Drosha-deficient mice and require fine-tuned TGFβ and TCR signaling for normal development. S1 cells begin to upregulate the chemokine receptor CCR7, which promotes migration to the medulla and is detectable on the surface of recent thymic emigrants (RTEs). S2 cells exit the thymus and travel to peripheral tissues, whilst S3 cells recirculate back into the thymus and remain resident for extended periods of time. Individual miRNAs acting on distinct developmental transitions and their targets are indicated. Double positive (DP), Stage 0 (S0), Stage 1 (S1), Stage 2 (S2), and Stage 3 (S3).

Developing iNKT cells display a dynamic miRNA expression profile substantially different from conventional T cells. Deletion of Dicer or Drosha in lymphocytes or T cells results in near complete absence of iNKT cells (20, 45, 46). miRNA-deficiency caused cell-intrinsic early stage blocks in development (Figure 1). Residual iNKT cells displayed reduced cytokine production and upregulation of CD69 following stimulation with the prototypical antigen α-GalCer, which argued that miRNA deficiency leads to a defect in iNKT TCR signaling (45, 46). An unbiased approach to identify miRNAs responsible for the developmental defect in Dicer-deficient iNKT cells was used, by comparing transcriptional signatures from Dicer-deficient iNKT cells to S1–2 and S3 WT iNKT cells. Matching differentially regulated Dicer-dependent genes with *in silico* predicted miRNA targets suggested several miRNAs to play a role in iNKT-cell development, such as miR-181 and members of the miR-17~92 cluster (47).

**Figure 2:**
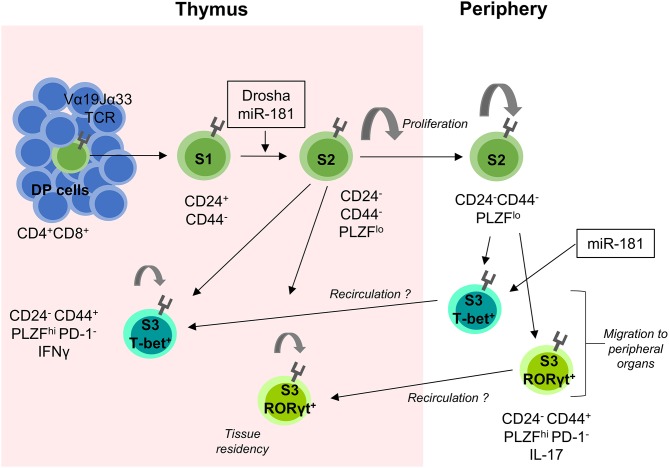
miRNA-mediated control of MAIT-cell development. MAIT cells are selected on MR1-expressing DP thymic cells. They proceed through several stages of development (S1–S3) as indicated by their surface expression of CD24 and CD44. The transition between S1 and S2 is heavily dependent on the presence of miRNAs as shown in Drosha-deficient mouse models. S2 MAIT cells begin to proliferate and develop into S3 effector MAIT cells defined by their expression of the transcription factors T-bet and RORγt. S2 cells can also exit the thymus, where they continue to proliferate and develop into S3 MAIT cells and migrate to peripheral tissues. It is postulated that some extra-thymically matured MAIT cells recirculate back to the thymus where they contribute to the thymic microenvironment and remain resident for extended periods of time. miR-181 is currently the only miRNA shown to be involved in MAIT cell development as indicated. MHC-related protein 1 (MR1), Double positive (DP), Stage 1 (S1), Stage 2 (S2), and Stage 3 (S3).

Similar to iNKT cells, development of MAIT cells is severely perturbed in the absence of miRNAs (20). In thymi of T-cell specific Drosha-deficient mice, MAIT-cell development was mostly arrested at S1 (20) (Figure 2).

Deletion of Dicer or Drosha results in defective Treg-cell development and function. T-cell specific deletion of miRNAs results in substantially reduced Treg-cell numbers in the thymus (48). In addition, residual miRNA-deficient Treg cells showed a reduced suppressive capacity and diminished homeostatic potential (48–50). Treg-cell specific ablation of miRNAs phenocopies loss of Foxp3, resulting in fatal multi-organ autoimmunity (50).

Taken together, developmental and functional defects in unconventional T cells in pan-miRNA deficient models highlight the critical role of miRNA-mediated post-transcriptional control in these cell types. However, the function of individual miRNAs and the corresponding underlying molecular mechanisms are only beginning to emerge.

Few miRNAs have shown to be critically involved in early conventional T-cell development, while agonist-selected T cell lineages have been demonstrated to be quite sensitive to miRNA-mediated regulation. Some of the more notable miRNAs that have been found to modulate developmental processes in unconventional T cell populations are detailed below from an iNKT-cell centric perspective (Figures 1, 2).

### Let-7

The let-7 family of miRNAs is the most abundant family of miRNAs in the genome and they play an important role in iNKT-cell development. Fetal hematopoietic cells express the LIN28A and LIN28B proteins which bind to let-7 precursor molecules, promoting their degradation, and preventing the biogenesis of functional let-7 miRNAs. This repression of functional let-7 miRNA expression shapes fetal lymphopoiesis (51). Exploiting this knowledge, transgenic expression of LIN28 has been employed to cause let-7 loss-of-function (52). *Vice versa*, inducible ectopic expression of mutated let-7 lacking binding sites for LIN28 results in let-7 gain-of-function (52). Analysis of these models provided evidence that dynamic upregulation of endogenous let-7 expression throughout thymic iNKT-cell differentiation results in a downregulation of PLZF, through direct targeting of *Zbtb16* mRNA (Figure 1). Let-7-dependent reduction in PLZF expression limits development of iNKT cells, whereas loss of function of let-7 rescued iNKT-cell development in *Zbtb16*-haploinsufficient mice. In addition, let-7 directs iNKT cells to terminally differentiate into IFNγ-producing NKT1 cells. In contrast, inhibiting the upregulation of let-7 miRNAs allowed iNKT cells to maintain high PLZF levels and favored the development of IL-4-producing NKT2 and IL-17-producing NKT17 cells. Dynamic upregulation of let-7 miRNAs during iNKT-cell development was triggered by exogenous stimuli in the thymic medulla, including IL-15, vitamin D, and retinoic acid (52). Given the requirement for PLZF in MAIT-cell development, it can be hypothesized that let-7 miRNAs may play a role in the generation of this cell lineage as well. Moreover, analysis of MAIT-cell development upon dysregulation of let-7 function may shed light on the relevance of differences in PLZF expression dynamics during iNKT- and MAIT-cell development.

### miR-17~92 and Its Paralogs miR-106b~25, and miR-106a~363

Comparisons between WT and Dicer-deficient developing iNKT cells revealed an increase in the expression of TGFβ receptor II (TGFβRII) mRNA in the absence of miRNA (47). TGFβRII is a subunit of the heterodimeric receptor for TGFβ, a cytokine known to protect against clonal deletion and promote the development of iNKT cells and other agonist-selected lineages (53–56). During S1 of iNKT-cell differentiation, TGFβ signaling through TGFβRII sustains iNKT-cell expansion. Upon progression through development S2 iNKT cells downregulate TGFβRII, which is a requirement for normal progression to S3. In Dicer-deficient mice, lack of regulation by miRNAs led to unconstrained TGFβRII expression and therefore TGFβ signaling, as well as an accumulation of early stage iNKT cells (47). The miR-17~92 cluster was experimentally validated to have two target sites in the *Tgfbr2* 3′UTR, which coincided with an inverse relationship between the expression of miR-17~92 miRNAs and TGFβRII. miR-17~92 comprises 6 miRNAs of 4 families. In addition, the cluster has two paralogs, miR-106a-363 and miR-106b~25, with different expression profiles. miR-17~92 is the predominant paralog in hematopoietic cells and its loss results in impaired B-cell and T-cell development (57, 58). Compound loss of additional paralogs results in more severe defects and individual targeting of members of the miR-17~92 cluster has provided a better understanding of miR-17~92 function (59).

Analysis of mice deficient in miR-17~92 and its paralogs revealed an increase in TGFβRII expression and therefore TGFβ signaling, while simultaneously demonstrating early iNKT cell defects at S2 (Figure 1). Interestingly, double-deficiency of Dicer and TGFβRII did not rescue frequencies or numbers of iNKT cells but appeared to aid cells stuck at S2 to differentiate into S3. Frequencies of S1 iNKT cells remained the same in Dicer and Dicer–TGFβRII double-deficient mice, revealing that potential regulation of TGFβRII expression by miR-17~92 cluster miRNAs promotes progression from S2 to S3 (47). Notably, both Dicer deficiency and miR-17~92 family deficiency resulted in a virtual absence of NKT17 cells (47). Whether miR-17~92 and/or TGFβ signaling contribute to MAIT-cell development remains elusive. In contrast, TGFβ signaling is pivotal for induction of Treg cells from naive precursors (56, 60) and may also contribute to Treg-cell development in the thymus (61–64). However, there is no direct evidence for a mechanistic link between miR-17~92 and TGFβ signaling in either of these processes. Rather, the role of miR-17~92 in Treg cells appears to be more complex. miR-17~92 has been implicated in regulation of IL-10 production by Treg cells with loss of miR-17~92 resulting in reduced suppressive capacity (65). By contrast, loss of miR-17 alone resulted in enhanced suppressive capacity, presumably due to de-repression of critical co-factors of Foxp3 (66). Thus, the role of miR-17~92 family miRNAs in Treg-cell development and function remains to be fully understood. It is particularly noteworthy that mice with deletion of miR-17~92 in all T cells are more resistant, whereas mice with Treg cell specific deletion of miR-17~92 are more susceptible to experimental autoimmune encephalitis when compared to WT mice (65, 66). These findings indicate diverging roles of this miRNA cluster prior to and after Foxp3 expression in Treg cells, suggesting major differences in induced and thymus-derived Treg cells with regard to miR-17~92 function.

### miR-150

miR-150 is dynamically expressed during development of iNKT cells with lowest expression at S1 followed by progressively increased expression through to S3. miR-150-deficiency resulted in an overall decrease in thymic iNKT cells, appearing to have a mild defect in the transition from S2 to S3, despite normal levels of S1 iNKT cells (67, 68) (Figure 1). Ubiquitous ectopic expression of miR-150 also resulted in an overall reduction in iNKT cells, displaying a developmental block between S1 and S2, through increased numbers of S1 iNKT cells (67). Perhaps an explanation for the similar phenotypes between miR-150-deficiency or transgenic ectopic expression lies in the need for specific constraints in miR-150 expression at precise stages of iNKT-cell development. For example, it could be the case that early iNKT cells require low levels of miR-150, explaining the normal progression of iNKT cells to S2 in miR-150-deficient mice followed by a block in further development due to a potential requirement for elevated levels of miR-150. Conversely, transgenic overexpression of miR-150 is likely to result in constitutively upregulated miR-150 at each iNKT-cell developmental stage, which may downregulate genes or pathways that are needed to move through development normally. If low expression of miR-150 was a requirement during early iNKT-cell development, then this would explain the block seen in miR-150-transgenic mice. Alternatively, huge proliferative expansion of iNKT cells in the early stages of development (69) could also explain the larger reduction in iNKT-cell numbers in miR-150-transgenic mice. Furthermore, increased production of IFNγ by splenic iNKT cells in miR-150-deficient mice (68), may be a result of skewed iNKT differentiation toward the iNKT1 subset. C-myb was identified as a potential target of miR-150 (67, 68, 70), with heterozygous c-myb-deficiency showing a reduction in splenic iNKT cells in mixed bone marrow chimeras, similar to transgenic expression of miR-150 (67). Further evidence is required to confirm the relationship between c-myb and miR-150 in the regulation in iNKT cells. This combined deficiency and overexpression data suggests that a dynamic and tightly regulated expression of miR-150 is required for normal iNKT-cell development. Recently, miR-150 has also been implicated in the peripheral differentiation and homeostasis of IELs (71). Mechanistically, it was suggested that miR-150, via inhibition of c-myb, limited expression of miR-20. In turn, reduced expression of miR-20 allowed for sufficient levels of TGFβ receptors to be expressed. Thus, miR-150-deficient IELs had reduced levels of TGFβ receptor. Notably, numbers of γδ IELs were most drastically reduced in the absence of miR-150. Despite the role of miR-150 in peripheral IELs, their intrathymic development was independent of miR-150. As the intrathymic generation of IELs is also controlled by TGFβ, this finding suggests that regulation of TGFβ signaling by miR-150 is context-dependent.

### miR-155

miR-155 was shown to have a mild effect on iNKT-cell development. Germline deletion of miR-155 shows relatively normal development and function of iNKT cells (72). However, transgenic overexpression of miR-155 resulted in a substantial block in development at S2, coupled with a reduction of the peripheral iNKT cell compartment (73). miR-155 expression was identified to progressively decline throughout iNKT-cell development, ending with the lowest expression in S3 cells (73). This suggests that tightly controlled miR-155 expression is required for normal iNKT-cell development. The Ets1 and ITK transcripts, modulators of TCR signaling and maturation, respectively, were brought forth as potentially relevant targets of miR-155 via *in vitro* luciferase assays. Mouse models deficient in Ets1 and ITK show a correlation in iNKT-cell developmental phenotypes to miR-155 transgenic mice (74–76). Interestingly, miR-155 transgenic mice showed upregulation of both targets in S1 and S2 cells, followed by a downregulation in S3, suggesting that increased expression of miR-155 in the earlier stages of iNKT-cell development does not render Ets1 and ITK susceptible to miR-155-mediated control (73).

In addition, miR-155 is the major miRNA shown to play a role in Treg-cell development. Foxp3 binds to the promoter and directly regulates the expression of miR-155's host gene *Bic*, which does not encode a functional protein (77). Impaired fitness coupled with inferior Foxp3 expression and stability was observed upon deletion of miR-155 (78). miR-155-deficient mice show reduced Treg-cell numbers (79) and upregulated suppressor of cytokine signaling 1 (SOCS1) (78). SOCS1 is a negative regulator of STAT5, a key downstream effector of IL-2R signaling, which is consequently diminished in miR-155-deficient mice (78, 80). It has been suggested that during Treg-cell development, induction of Foxp3 drives high expression of miR-155, maintaining the competitive fitness of Tregs (78). The miR-155/SOCS1 axis in Treg cells is one of only a few examples of rigorous analysis into the contribution of a single mRNA target to the overall function of miRNA-mediated regulation. To this end, a mutation in the *Socs1* 3′UTR was introduced to disrupt the interaction between *Socs1* mRNA and miR-155 and thus relieving SOCS1 from miR-155-mediated control (81). This analysis showed that some effects exerted by miR-155 were directly attributable to SOCS1, such as Treg-cell homeostasis, but others such as regulation of antiviral immunity, were not. Overall, regulatory functions exerted by miR-155 highlight the context-dependency of miRNA action.

### miR-181

Six members of the miR-181 family have been identified in mice and humans. The family is divided into three mini-clusters, miR-181a/b-1, a/b-2, and c/d, each of which is encoded on a separate chromosome. Mature miR-181a-1 and a-2 as well as b-1 and b-2 share an identical sequence. Mature miR-181a-1 and b-1 are encoded by murine chromosome 1 and are situated ~150 bp apart. High expression of the miR-181a/b-1 cluster is observed in the thymus, accounting for ~98% of all the miR-181 species (82).

miR-181a/b-1 is dynamically expressed throughout thymocyte development and accounts for the largest enrichment of a miRNA family at any stage of development (83). This enrichment occurs at the DP stage, during which thymocytes undergo selection (83, 84). Transcripts containing predicted seed matches to miR-181 family members are depleted at the DP stage and are contrastingly enriched at the DN3 stage of development (83). So far, miR-181a/b-1 has been implicated in TCR signaling and thymocyte selection during these developmental stages (82, 85–87).

TCR signaling requires a host of coordinated phosphorylation and dephosphorylation events within a network of intracellular signaling molecules. T cells express over 40 different negative regulators of TCR signaling including multiple phosphatases for each of the fundamental TCR signaling kinases, such as Lck, Zap70, and ERK (88). For a cell to tune its internal selection or activation thresholds, it must simultaneously modulate multiple signaling molecules with minimal sequence homology. In 2007, Li et al. (85) identified miR-181a as modulator of TCR sensitivity by controlling multiple intracellular signaling molecules. Only modest regulation of the phosphatases PTPN22, SHP-2, and DUSP6 by miR-181a was required to yield substantial differences in both activation and selection thresholds. Using retroviral overexpression, negative regulation of these phosphatases by miR-181a was confirmed via multiple methods including luciferase assay for target validation, and western blot and qPCR for visualization of reduced protein and mRNA levels, respectively, in the presence of increased miR-181a. An increase in both Lck and ERK phosphorylation could be seen in the presence of increased miR-181a (85), which is consistent with their reported dephosphorylation by PTPN22 and DUSP5/6, respectively (89, 90). Moreover, restoring DUSP6 expression to normal levels by co-expressing miR-181a and *Dusp6* mRNA with mutated miR-181a target sites, reduces basal Lck serine phosphorylation to the background level (85). In addition to miR-181a overexpression studies, Li et al. also performed loss-of-function analyses using miR-181a antagomirs. Simulating thymocyte development *in vitro* using fetal thymic organ culture in the presence of miR-181a antagomirs and TCR antigens revealed a large reduction in negative selection and an impairment of positive selection, suggesting that miR-181a sets a threshold for selection in the thymus. Upon antagomir treatment in DP thymocytes, both mRNA and protein levels of PTPN22, SHP-2, DUSP5, and DUSP6 were elevated, along with a decrease in ERK activation. Therefore, miR-181a was identified as an intrinsic modulator of TCR sensitivity through targeting multiple negative regulators of TCR signaling providing an *in vitro* framework for the mechanism behind miR-181a's involvement in thymocyte selection. However, the role of miR-181a in selection of conventional T cells *in vivo* appears to be complex and remains incompletely understood (86).

In contrast, miR-181 is critical for development of unconventional T cells, most notably iNKT and MAIT cells, but also Treg cells. Loss of miR-181a/b-1 resulted in a massive reduction in the iNKT cell compartment due to a block in the transition between S0 and S1, coupled with a reduced proliferation capacity (Figure 1). Consistent with the previously suggested function of miR-181 as modulator of TCR signaling, miR-181a/b-1-deficient thymocytes also displayed a decreased capacity to transmit signals through the TCR and iNKT-cell development was rescued *in vivo* through administration of supraphysiological levels of cognate α-GalCer ligand (82). A Vα14-transgenic TCR was also shown to rescue the defect in iNKT-cell development in miR-181a/b-1-deficient mice (91). An independent study characterizing miR-181a/b-1-deficient mice suggested that the deficiency in iNKT-cell development was rather due direct targeting of *Pten*, suggesting that miR-181a/b-1 constitutes a metabolic rheostat during T-cell development (92). Although haploinsufficiency of *Pten* was able to partially rescue miR-181a/b-1-induced iNKT-cell development, another study showed that rescue by a Vα14 TCRα transgene did not alter PTEN levels (91). Although a dual function of miR-181a/b-1 for iNKT-cell development is conceivable, this finding suggested that targeting PTEN is most likely secondary over modulation of TCR signaling. Nevertheless, open questions concerning the discrepancies between phenotypes of the two independent mouse models persist.

Certain populations of γδT cells also depend on strong TCR signals for development. Surprisingly, γδT-cell development was largely independent of miR-181a/b-1 and found to be relatively normal in the absence of miR-181a/b-1 (82, 93). However, an increase in the liver γδ NKT cell compartment was observed, attributable to homeostatic expansion in the absence of αβ iNKT cells (93). The pool of γδ NKT cells in the liver of miR-181a/b-1-deficient mice showed a reduced capacity to secrete IFNγ, perhaps hinting to a yet undefined role of miR-181 in IFNγ regulation of CD27^+^ γδT cells. Recent data show that both miR-181a and miR-181d are upregulated in CD27^+^ compared to CD27^−^ cells, which points toward a possible role in γδT-cell differentiation (93). In regard to this, it is important to note that normal miR-181d expression levels are present in miR-181a/b-1-deficient mice and this could have an obscuring effect on miR-181a-deficiency.

MAIT cells can partially compensate for loss of iNKT cells, which is exemplified in the absence of CD1d. Alternatively, the many parallels in development of both unconventional T cell subsets might extend to a critical role for miR-181a/b-1 in MAIT-cell development as well, although it remains to be shown whether MAIT-cell selection also depends on strong TCR signals (41). Indeed, formation of MAIT cells was critically impaired in the absence of miR-181a/b-1 with a partial developmental block at S1 and severely limited proliferation of S2 cells (94) (Figure 2). As a consequence, residual MAIT cells in miR-181a/b-1-deficient mice failed to upregulate the key transcription factor PLZF. Notably, loss of miR-181a/b-1 results in a *bona fide* phenocopy of defective MAIT-cell development in pan-miRNA-deficient mice (20). Although loss of miR-181a/b-1 completely precluded intrathymic maturation of MAIT cells, some peripheral MAIT cells acquired PLZF and the MAIT1 transcription factor T-bet and to a much lesser extent the MAIT17 transcription factor RORγt. These findings highlight the capacity of MAIT cells for extra-thymic terminal maturation. *Bona fide* MAIT-cell development was rescued by retrogenic expression of the invariant Vα19 TCRα chain, providing indirect evidence for agonist selection.

**Figure 3:**
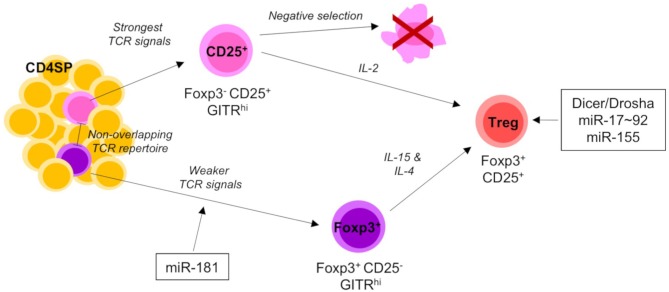
miRNA-mediated control of Treg-cell development. Thymic-derived Treg-cell development begins at the CD4 SP stage of development and proceeds via one of two different pathways. The first Treg precursors to arise are the CD25^+^ population. CD25^+^ precursors require strong TCR signals and IL-2 signaling to mature into CD25^+^Foxp3^+^ Tregs. The alternative pathway of Treg-cell development is characterized by the generation of Foxp3^+^ precursors. Foxp3^+^ precursors develop from comparably weaker TCR signals to CD25^+^ precursors and share little TCR repertoire overlap. As they mature into Tregs they upregulate CD25 and require both IL-4 and IL-15 signaling. Mature Tregs emigrate from the thymus to join the peripheral T cell pool. Total (Dicer/Drosha) and individual miRNAs acting on distinct developmental transitions are indicated. Single positive (SP) and T cell receptor (TCR).

Use of a *Rag*^GFP^ molecular timer to label freshly developing cells in the thymus, revealed a substantial defect in development of Treg cells in the absence of miR-181a/b-1 due to reduced TCR signaling (95) (Figure 3). Treg cells are generated from two different precursors. Foxp3^−^CD25^+^ precursors receive strong TCR signals, whilst Foxp3^+^CD25^−^ precursors are characterized by a history of somewhat weaker TCR signals (5, 36). The former are virtually unaffected by loss of miR-181a/b-1, suggesting that TCR signals in these precursors are either not sufficiently dampened or that this subset is replenished by thymocytes otherwise marked for clonal deletion. In contrast, formation of Foxp3^+^CD25^−^ precursors is substantially delayed. Together, these data imply that either Foxp3^+^CD25^−^ precursors constitute the major source of thymus-derived Treg cells or that loss of miR-181a/b-1 imposes an additional block in Treg-cell development at the transition between Foxp3^−^CD25^+^ precursors and mature Treg cells. Interestingly, loss of miR-181a/b-1 allowed for the generation of a numerically normal peripheral Treg-cell compartment through homeostatic expansion. In consequence, these Treg cells had a limited TCR diversity. Notably, peripheral murine Treg cells express very low levels of miR-181a/b-1. Nevertheless, peripheral miR-181a/b-1-deficient Treg cells expressed elevated levels of CTLA-4 resulting in an increased suppressive capacity. The molecular mechanism resulting in elevated expression of CTLA-4 remains in part elusive.

In contrast to other miRNAs discussed so far, which display highly context-dependent functions in unconventional T cells, miR-181 as rheostat of TCR signaling has emerged as unifying master regulator of unconventional T cells. Of note, development of IELs remains ill-characterized with regard to regulation by miRNAs. However, the recent identification of more clearly defined intrathymic IEL precursors will be of substantial advantage for such an analysis (31).

## Regulation of miRNA Function Beyond Dynamic Expression

T cells express over 600 different miRNAs, with individual miRNA expression levels differing as much as 1,000-fold throughout T-cell development. Dynamic mRNA composition as T cells advance through development, alters the availability of miRNA recognition sites and therefore the miRNA “targetome” (84). Thus, many miRNAs may target one mRNA, while one miRNA may target many mRNAs. Furthermore, recent reports suggest that miRNAs exhibit different targeting hierarchies in different cell types (96). Some striking examples of such dependency on cellular context have been highlighted above. However, recent evidence suggests that dynamic and/or high expression levels do not necessarily indicate functional relevance of a given miRNA.

miR-21 is prominently expressed in immature DN thymocytes followed by a massive decline toward more mature developmental stages (84, 97). Expression levels are high again in iNKT cells with miR-21 being the most highly upregulated when compared to conventional T cells, prompting it to be a potential candidate for iNKT-cell regulation (45). However, neither deletion nor its overexpression in mixed bone-marrow chimeric mice revealed a role of miR-21 for both conventional and unconventional T-cell development, including iNKT cells, Treg cells, and γδT cells (97). The underlying mechanism for this apparent discrepancy between high levels of expression vs. lack of functional relevance remains elusive. A study comparing primary liver cells with cancer cells revealed that in primary cells miR-21 was largely unable to repress its predicted targets (98). This failure in repressive function was associated with limited recruitment to polysomes, whereas recruitment of miR-21 to polysomes was substantial in cancer cells, suggesting that the weak silencing capacity of miR-21 in primary cells is a consequence of selective redistribution of its target mRNAs away from polysomes.

Another recent study revealed that most cells contain reservoirs of low molecular weight RISCs (LMW-RISC) that are not associated with mRNAs and are therefore inactive (99). Upon T-cell activation, signal transduction pathways were demonstrated to increase the assembly of high molecular weight RISCs (HMW-RISC), which prompted miRNA-mediated repression, despite there being no net increase in miRNA expression (99). Interestingly, in both conditions miR-21 was preferentially detected in LMW-RISC, which is consistent with its limited repressive function. Another mechanism of post-transcriptional regulation of miRNA has recently been demonstrated to be 5′-end phosphorylation (100). This study showed that only 5′-end phosphorylated miR-34 was incorporated into RISCs and phosphorylation of mature, yet inactive miR-34, was dependent on DNA damage.

Overall, these studies highlight mechanisms of regulation of miRNA function beyond dynamic changes in expression. Understanding post-transcriptional modification of miRNAs is pivotal for a complete understanding of miRNA-mediated regulation in development and function of unconventional T cells.

## Author Contributions

SW and AK wrote the manuscript.

### Conflict of Interest

The authors declare that the research was conducted in the absence of any commercial or financial relationships that could be construed as a potential conflict of interest.
